# Transstadial immune activation in a mosquito: Adults that emerge from infected larvae have stronger antibacterial activity in their hemocoel yet increased susceptibility to malaria infection

**DOI:** 10.1002/ece3.5192

**Published:** 2019-04-23

**Authors:** Lisa D. Brown, Lillian L. M. Shapiro, Grayson A. Thompson, Tania Y. Estévez‐Lao, Julián F. Hillyer

**Affiliations:** ^1^ Department of Biological Sciences Vanderbilt University Nashville Tennessee; ^2^ Present address: Department of Biology Georgia Southern University Statesboro Georgia

**Keywords:** *Anopheles gambiae*, Culicidae, hemocyte, immunity, insect, malaria, metamorphosis

## Abstract

Larval and adult mosquitoes mount immune responses against pathogens that invade their hemocoel. Although it has been suggested that a correlation exists between immune processes across insect life stages, the influence that an infection in the hemocoel of a larva has on the immune system of the eclosed adult remains unknown. Here, we used *Anopheles gambiae* to test whether a larval infection influences the adult response to a subsequent bacterial or malaria parasite infection. We found that for both female and male mosquitoes, a larval infection enhances the efficiency of bacterial clearance following a secondary infection in the hemocoel of adults. The adults that emerge from infected larvae have more hemocytes than adults that emerge from naive or injured larvae, and individual hemocytes have greater phagocytic activity. Furthermore, mRNA abundance of immune genes—such as cecropin A, Lysozyme C1, Stat‐A, and Tep1—is higher in adults that emerge from infected larvae. A larval infection, however, does not have a meaningful effect on the probability that female adults will survive a systemic bacterial infection, and increases the susceptibility of females to *Plasmodium yoelii*, as measured by oocyst prevalence and intensity in the midgut. Finally, immune proficiency varies by sex; females exhibit increased bacterial killing, have twice as many hemocytes, and more highly express immune genes. Together, these results show that a larval hemocoelic infection induces transstadial immune activation—possibly via transstadial immune priming—but that it confers both costs and benefits to the emerged adults.

## 1 INTRODUCTION

For decades, the classical assumption was that invertebrate immune systems are not adaptive and respond identically to multiple infections (Beckage, 2008); however, studies on diverse groups of insects show that the innate immune system varies in response to repeated challenges (Bartholomay & Michel, 2018; Cooper & Eleftherianos, 2017; Hillyer, 2016; Masri & Cremer, 2014; Melillo, Marino, Italiani, & Boraschi, 2018; Milutinovic, Peuss, Ferro, & Kurtz, 2016; Shaw et al., 2018). Most notably, a prior infection can provide an insect with partial or full protection from a subsequent infection, with this increased protection being due to (a) a recall response that is faster and more powerful, (b) a shift from one type of response to another, and/or (c) a sustained immune response (Hamilton, Siva‐Jothy, & Boots, 2008; Melillo et al., 2018). Although many studies on this topic indicate that protection lasts the entirety of a single life stage, there is limited evidence that immune system activation persists across molts (Thomas & Rudolf, 2010).

Metamorphosis involves considerable shifts in the ecology and physiology of many invertebrates, including alterations to the immune system (League, Estevez‐Lao, Yan, Garcia‐Lopez, & Hillyer, 2017; League & Hillyer, 2016; Russell & Dunn, 1996). In mosquitoes, metamorphosis has decoupled the larval and adult immune systems such that immunity is strongest in larvae and declines after eclosion and with adult age (League et al., 2017). However, multiple reports have demonstrated that the conditions experienced by mosquito larvae can influence adult immune traits (Araujo, Gil, & e‐Silva, 2012; Moller‐Jacobs, Murdock, & Thomas, 2014; Murdock, Paaijmans, Cox‐Foster, Read, & Thomas, 2012; Muturi, Kim, Alto, Berenbaum, & Schuler, 2011; Suwanchaichinda & Paskewitz, 1998; Telang, Qayum, Parker, Sacchetta, & Byrnes, 2012; Vantaux, Ouattarra, Lefevre, & Dabire, 2016). For example, larval nutritional stress results in adults that have decreased melanization activity, fewer hemocytes (immune cells), and higher expression of immune effector genes (Muturi et al., 2011; Suwanchaichinda & Paskewitz, 1998; Telang et al., 2012). Moreover, environmental exposure of *Aedes aegypti* larvae to different bacteria impacts the relative strength of the antimicrobial response of the resultant adults (Dickson et al., 2017; Moreno‐Garcia, Vargas, Ramirez‐Bello, Hernandez‐Martinez, & Lanz‐Mendoza, 2015). For example, the presence of *Escherichia coli* during larval development increases phenoloxidase activity, nitric oxide production, and antibacterial activity in adults (Moreno‐Garcia et al., 2015), whereas inhabiting a larval environment containing an Enterobacteriaceae isolate decreases antibacterial activity in adults (Dickson et al., 2017). Additionally, in both *Ae. aegypti* and *Anopheles gambiae*, the gut microbe *Chromobacterium* sp. has both larvicidal and adulticidal activity, but the mosquitoes that survive to adulthood are more resistant to dengue virus and *Plasmodium* parasites, respectively (Ramirez et al., 2014). The mechanisms that drive these effects are largely unknown, but for holometabolous insects, it has been suggested that a correlation exists between immune processes across life stages (Fellous & Lazzaro, 2011).

**Figure 1:**
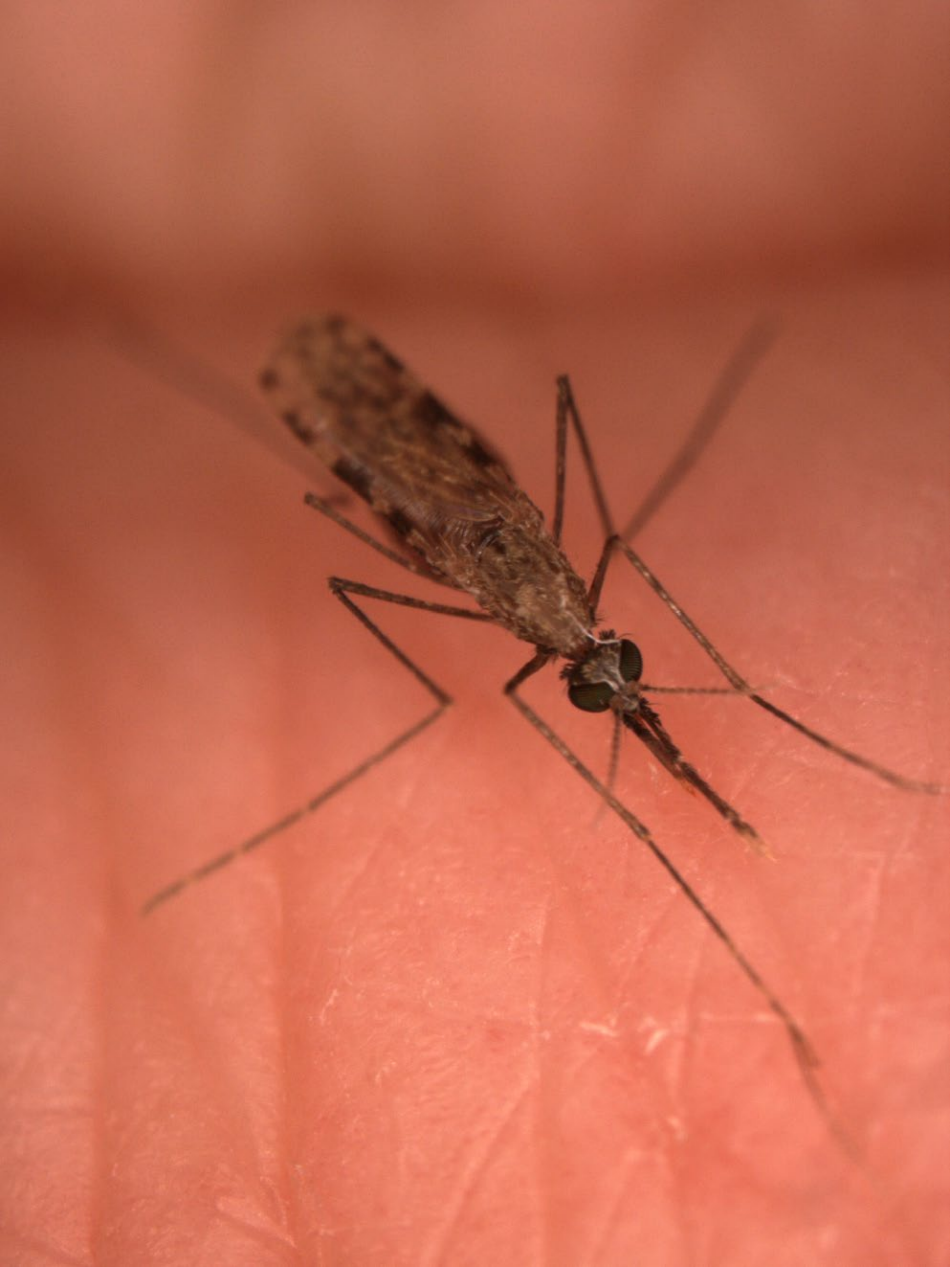
The African malaria mosquito, *Anopheles gambiae*

The aquatic habitats of mosquito larvae are rife with microorganisms that can invade the hemocoel (body cavity) (Bartholomay & Michel, 2018; Granados, 1980; Kalucy & Daniel, 1972; Petersen, Chapman, & Woodard, 1968; Sweeney, Inman, Bland, & Wright, 1983; Washburn, Egerter, Anderson, & Saunders, 1988; Yassine, Kamareddine, & Osta, 2012). Consequently, mosquito larvae mount powerful cellular and humoral immune responses against pathogens in their hemocoel (Biron et al., 2005; Dimopoulos, Richman, Muller, & Kafatos, 1997; Duncan et al., 2012; Kalucy & Daniel, 1972; League et al., 2017; League & Hillyer, 2016; Meredith, Hurd, Lehane, & Eggleston, 2008; Richman et al., 1996; Shin et al., 2005). Recently, we demonstrated that a larval‐acquired infection in the hemocoel is transstadially transmitted to the hemocoel of an adult, which may have important implications for adult immunity (Brown, Thompson, & Hillyer, 2018). We hypothesize that a larval‐acquired infection activates the immune system such that the resultant adult is better able to respond to a future infection. In the present study, we investigated whether immune activation in larvae of the African malaria mosquito, *An. gambiae* (Figure 1), influences the ability of adult mosquitoes to combat an infection. We found that a larval bacterial infection increases the ability of mosquitoes to kill bacteria acquired as adults, but decreases the ability to control a malaria infection. Additionally, a larval infection increases the number of circulating hemocytes in the eclosed adults, enhances the phagocytic activity of individual hemocytes, and increases the expression of immune genes. However, a larval infection does not have a meaningful effect on the ability of female adults to survive a bacterial infection.

## 2 MATERIALS AND METHODS

### 2.1 Mosquito rearing and maintenance

*Anopheles gambiae* (G3 strain) were reared and maintained in an environmental chamber at 27°C, 75% relative humidity, and a 12 hr:12 hr day:night cycle as previously described (Coggins, Estevez‐Lao, & Hillyer, 2012). Briefly, larvae were hatched in deionized water and fed a daily mixture of koi fish food and baker's yeast. Experimental larvae were treated in the early 4th instar stage, and after pupation and subsequent eclosion, adult mosquitoes were fed 10% sucrose ad libitum.

### 2.2 Mosquito injection and larval treatment groups

Early 4th instar larvae were injected in their hemocoel at the mesothorax using a Nanoject II Auto‐Nanoliter Injector (Drummond Scientific Company). Larvae received 69 nl of one of the following: (a) sterile Luria‐Bertani's rich nutrient medium (LB; injury group); (b) live, tetracycline‐resistant, GFP‐expressing *Escherichia coli* (modified DH5α) in LB; (c) heat‐killed *E. coli* in LB; (d) live *Enterobacter* sp. isolate Ag1 in LB (Jiang, Alvarez, Kukutla, Yu, & Xu, 2012); (e) live *S. aureus* (RN6390) in tryptic soy broth; or (f) 0.2% solids 1‐μm‐diameter red fluorescent (580/605) carboxylate‐modified microspheres (Invitrogen) in phosphate‐buffered saline (PBS). An additional group of larvae did not receive an injection (naïve). As 5‐day‐old adults, all mosquitoes were injected at the thoracic anepisternal cleft with 69 nl of live, tetracycline‐resistant, GFP‐expressing *E. coli* in LB, with the exception of one group related to the experiments presented in section 3.2.

### 2.3 Bacterial infection and measurement of bacterial infection intensity

In a shaking incubator, *E. coli* and *S. aureus* were grown overnight at 37°C, and *Enterobacter* sp. was grown overnight at 27°C. Infection doses were estimated prior to larval injections by measuring the optical density (OD_600_) of bacterial cultures using a BioPhotometer Plus spectrophotometer (Eppendorf AG). Absolute doses were determined by spreading a 1:1,000 dilution of the bacterial culture on an LB or tryptic soy agar plate, incubating the plate overnight at 37°C or 27°C (depending on the species), and then counting the resultant colony‐forming units (CFUs). Across all experimental trials, infection doses per mosquito averaged 34,109 (±542 *SEM*) for *E. coli* (OD_600_ = 5), 54,119 (±852 *SEM*) for *Enterobacter* sp. (OD_600_ = 0.01), and 27,347 (±472 *SEM*) for *S. aureus* (OD_600_ = 2). For experiments using heat‐killed *E. coli*, 200 μl of bacterial culture (OD_600_ = 5) was incubated at 95°C for 10 min on an IncuBlock heating block (Denville Scientific) and injected after cooling to room temperature. Plating of the heat‐killed culture resulted in no CFUs, which confirmed that all bacteria were dead prior to injections.

After larval treatments, mosquitoes were allowed to eclose and the resultant adults were injected with *E. coli* 5 days later. Mosquitoes were homogenized in PBS 24 hr after infection, diluted, and spread on LB agar plates containing tetracycline. Plates were incubated overnight at 37°C, and the number of CFUs was counted and used to calculate infection intensity (the mean number of *E. coli* in an infected mosquito). To confirm that all colonies originated from the *E. coli* inoculums, plates were also viewed by fluorescence microscopy to verify the expression of GFP. Three independent trials consisting of ≥10 mosquitoes per treatment group and sex were conducted, and the data were pooled for analysis.

### 2.4 Quantification of circulating hemocytes

Circulating hemocytes were collected by perfusion from 6‐day‐old adults at 24‐hr post‐treatment with *E. coli* as previously described (League et al., 2017). An additional group that did not receive an *E. coli* injection as adults was also analyzed. Briefly, an incision was made across the last abdominal segment, a microinjection needle was inserted into the neck membrane, and approximately 200 μl of Grace's insect medium was injected. The diluted hemolymph that exited through the abdominal incision was collected within a 1‐cm‐diameter etched ring on a Rite‐On glass slide (Gold Seal). After allowing the hemocytes to adhere to the slide for 20 min at room temperature, cells were fixed and stained using Hema 3 (Fisher Scientific), dried, and mounted under a coverslip using Poly‐Mount (Polysciences) (Hillyer, Schmidt, Fuchs, Boyle, & Christensen, 2005). The total number of hemocytes was then counted under bright‐field illumination at 40X magnification using a Nikon 90i compound microscope (Nikon, Tokyo, Japan). Three independent trials consisting of 15 individuals per treatment group and sex were conducted, and the data were pooled for analysis.

### 2.5 Quantification of phagocytosis by circulating hemocytes

Five‐day‐old adults were injected with live *E. coli*, and 1 hr later, the hemocytes were collected by perfusion. Hemocytes were allowed to adhere to the slide for 20 min at room temperature, fixed for 5 min by adding 50 μl of 4% formaldehyde and 0.03 mM Hoechst 33,342 (nuclear stain) in PBS, and coverslips were mounted with Aqua‐Poly/Mount. Hemocytes were examined under 100X magnification using simultaneous DIC and fluorescence illumination on a Nikon 90i compound microscope. For each mosquito, the number of bacteria that had been phagocytosed by each of the first 50 hemocytes viewed was recorded. These values were used to calculate two parameters: (a) the phagocytic index, which is defined as the percentage of cells that engage in phagocytosis; and (b) the phagocytic capacity, which is defined as the average number of phagocytosed bacteria per hemocyte (Coggins et al., 2012; Hillyer et al., 2005; League et al., 2017). Three independent trials consisting of five individuals per treatment group and sex were conducted, and the data were pooled for analysis.

### 2.6 Quantification of immunity gene expression by qPCR

The expression of select immune genes was measured in 5‐day‐old adults that emerged from naïve, injured, and *E. coli*‐infected larvae, using a protocol we have previously described (League et al., 2017). Briefly, for each treatment group the total RNA from the whole bodies of 10 mosquitoes was isolated using TRIzol reagent (Invitrogen). RNA was re‐purified using the PureLink Micro‐to‐Midi Total RNA Purification System (Invitrogen), treated with RQ1 RNase‐Free DNAse I (Promega), and then used as template for cDNA synthesis using an Oligo(dT)_20_ primer and the SuperScript III First‐Strand Synthesis System for RT‐PCR (Invitrogen). Real‐time quantitative PCR (qPCR) was performed using gene‐specific primers and Power SYBR Green PCR Master Mix (Applied Biosystems) on a Bio Rad CFX Connect Real‐Time PCR Detection System. Sequences and gene IDs were downloaded from the AgamP3 and AgamP4 assemblies of the *A. gambiae* (PEST) genome in www.vectorbase.org, and primer sequences are presented in Table 1. Relative quantification was performed using the 2^−ΔΔC^_ T_ method, and mRNA levels were calculated relative to the naïve group of each mosquito sex (Coggins et al., 2012; League et al., 2017; Livak & Schmittgen, 2001). Three independent trials were conducted, and each trial was analyzed in duplicate. Data are presented as the average fold‐change relative to the naïve group of each adult sex. The genes assayed were Cecropin A (*CecA* or *Cec1*; AGAP000693), Lysozyme C1 (*LysC1*; AGAP007347), Stat‐A (*Stat‐A*; AGAP010423), Thioester‐containing protein 1 (*Tep1*; AGAP010815), Prophenoloxidase 6 (*PPO6*; AGAP004977), and *RpS17* (AGAP004887; negative control). Ribosomal protein 7 (*RpS7*; AGAP010592) was used as the reference gene. The immune genes were selected because their expression is upregulated in larvae following infection (League et al., 2017).

**Table 1:** Gene names, VectorBase gene IDs, and primers used for quantitative RT‐PCR

Gene	VectorBase ID	Forward primer (5'→3')	Reverse primer (5'→3')	Transcript (bp)	Genomic (bp)
*Tep1*	AGAP010815	GACGTCCAAATACGGATCTCA	CTTTCAGGCATCACCCGTAT	184	Does not amplify
*Stat‐A*	AGAP010423	CCTTGGAACAGGAAGAGCTG	ACGACCTACCCGTGCACTAA	218	218
*CecA*	AGAP000693	GCTGAAGAAGCTGGGAAAGA	ATGTTAGCAGAGCCGTCGTC	158	247
*PPO6*	AGAP004977	AGAGCCACTACCGGAAGGAT	TCGATGCTCTCAGCAATACG	174	242
*LysC1*	AGAP007347	ACGGCATCTTCCAGATCAAC	CATTGCAGTGGTTCTTCCAG	180	259
*RPS17*	AGAP004887	GACGAAACCACTGCGTAACA	TGCTCCAGTGCTGAAACATC	153	264
*RPS7*	AGAP010592	GACGGATCCCAGCTGATAAA	GTTCTCTGGGAATTCGAACG	132	281

### 2.7 Mosquito survival

On the 5th day of adulthood, female and male mosquitoes that eclosed from naïve, injured, or *E. coli*‐infected larvae were infected with *E. coli*. Mosquito survival was then tracked for the next 15 days. Three independent trials were conducted—with each trial being composed of ≥29 adult females and ≥12 adult males per treatment—and the data were pooled for analysis.

### 2.8 Prevalence and intensity of *Plasmodium yoelii* infections

Female adults from each treatment were allowed to feed for 30 min on anesthetized C57BL/6 mice (>6 weeks old; >16 g; parasitemia = ~8%) infected with *Plasmodium yoelii yoelii* (clone 17XNL:PyGFP; obtained through BEI Resources, NIAID, NIH) (Ono, Tadakuma, & Rodriguez, 2007). After exposure to mice, unfed mosquitoes were removed and the blood‐fed mosquitoes were maintained in the environmental chamber described above. At 7–9 days postfeeding, midguts were resected and the number of oocysts was counted. Data were analyzed in terms of prevalence (percentage of mosquitoes that were infected) and intensity (number of oocysts in infected mosquitoes). Three independent trials were conducted—each trial comprising of ≥23 adult females per treatment. The data were pooled for analysis, with the sample sizes of larval naïve, injury and *E. coli*‐infected groups being 138, 137, and 137 mosquitoes, respectively (81, 94 and 102, respectively, when only considering mosquitoes that became infected).

### 2.9 Ethics statement on the use of animals

This study was carried out in accordance with the recommendations in the Guide for the Care and Use of Laboratory Animals of the National Institutes of Health, USA. The protocol was approved by Vanderbilt University's Institutional Animal Care and Use Committee (IACUC; protocol #M1800023). Animals were maintained in a certified animal room and were cared for by trained personnel and veterinarians.

### 2.10 Statistical analyses

All statistical analyses were performed using GraphPad Prism version 7 (GraphPad Software), and differences were considered significant at *p* ≤ 0.05. Data on bacterial clearance efficiency, total number of circulating hemocytes, phagocytic index, phagocytic capacity, and immune gene expression were analyzed using two‐way ANOVA, followed by Tukey's post hoc test. Two‐way ANOVA yields three distinct *p*‐values, which address (a) whether treatment affects the outcome, (b) whether sex affects the outcome, and (c) whether the effect of treatment varies as a function of sex (interaction). Data on *Plasmodium* infection—collected only in female mosquitoes because this is the only sex that drinks blood—were analyzed by the Kruskal–Wallis test followed by Dunn's multiple comparisons post hoc test. Pairwise comparisons of mosquito survival were conducted using the Logrank test.

## 3 RESULTS

### 3.1 Larval infection increases the ability of adult mosquitoes to kill bacteria

**Figure 2:**
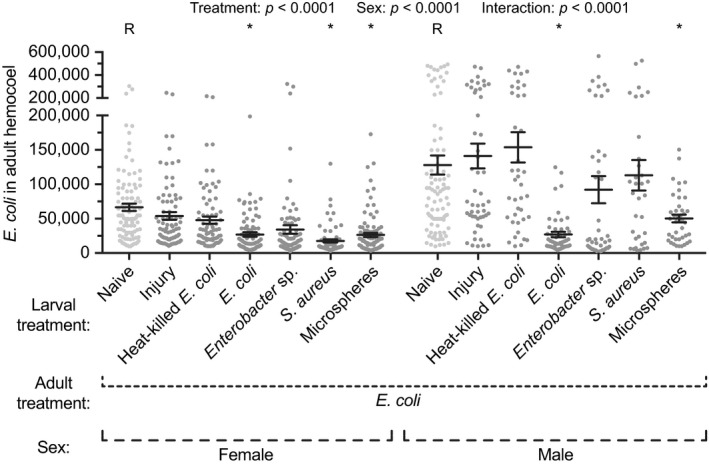
Infection intensity in adult female and male mosquitoes following various larval treatments. Larvae were left unmanipulated (naïve), injured, injected with heat‐killed *E. coli* or microspheres, or infected with *E. coli*, *Enterobacter* sp., or *S. aureus*. Five days after eclosion, the female and male adults were infected with *E. coli*, and the *E. coli* infection intensity was measured 24 hr later. Circles mark the data points, horizontal line marks the mean, whiskers denote the standard error of the mean, and asterisks denote significant differences (Tukey's *p* < 0.05) between the larval naïve group of a given sex (R) and a larval treatment

To determine whether a larval immune challenge alters the outcome of an infection acquired during the adult stage, early 4th instar larvae received one of various treatments, and five days after eclosion, the adults were infected with *E. coli* and the infection intensity in their hemocoel was measured 24 hr later. Infection intensity was altered by larval treatment (Figure 2; treatment *p* < 0.0001). Specifically, the infection intensity in female adults that emerged from larvae infected with *E. coli*, *Enterobacter* sp., or *S. aureus* was 60%, 49%, and 74% lower, respectively, than the infection intensity in female adults that eclosed from untreated larvae. Likewise, infection intensity in male adults that emerged from the same larval treatment groups was 79%, 28%, and 12% lower, respectively, than the infection intensity in male adults that arose from naïve larvae. Additionally, injecting larvae with microspheres, regardless of sex, resulted in adults that had infection intensities that were 61% lower than those in adult mosquitoes that emerged from untreated larvae. Injuring larvae or injecting them with heat‐killed *E. coli* had a negligible impact on an adult infection. Finally, mosquito sex impacted the infection intensity, with males having higher infection intensities than females (sex *p* < 0.0001). Thus, these data show that activation of an immune response at the larval stage, either via infection or via the introduction of an undegradable immune elicitor (microspheres), results in adults that are more proficient at killing bacteria in their hemocoel. This protection occurs regardless of whether the infection acquired as an adult is the same or different from the infection acquired as a larva.

### 3.2 An infection at the larval stage increases the number of circulating hemocytes of the resultant adult

Because hemocytes are the first cellular responders to a systemic infection (Hillyer & Strand, 2014), we hypothesized that a larval infection results in adults that have more hemocytes. To test this, larvae received one of various treatments, and five days after eclosion, the adults were infected with *E. coli* and the number of circulating hemocytes was counted 24 hr later. To determine whether differences were due to an induced response at the adult stage or a response carried over from the larval stage, we counted the number of hemocytes in a group of unmanipulated adults that had been infected with *E. coli* as larvae.

**Figure 3:**
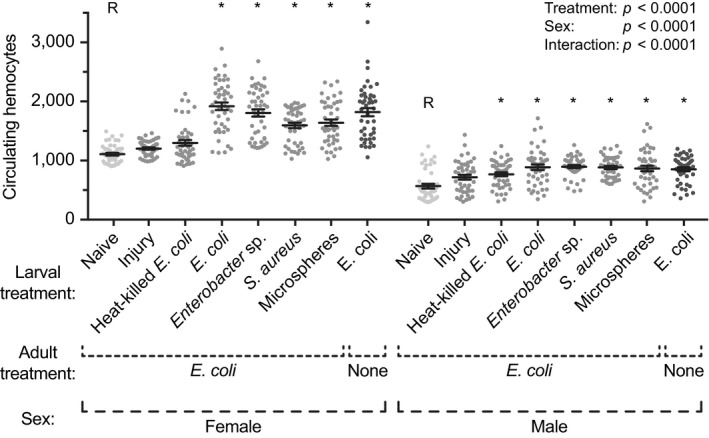
Number of hemocytes in adult female and male mosquitoes following various larval treatments. Larvae were left unmanipulated (naïve), injured, injected with heat‐killed *E. coli* or microspheres, or infected with *E. coli*, *Enterobacter* sp., or *S. aureus*. Five days after eclosion, the female and male adults were infected with *E. coli*, and the number of hemocytes was counted 24 hr later. An additional group was infected with *E. coli* as larvae but left untreated as adults. Circles mark the data points, horizontal line marks the mean, whiskers denote the standard error of the mean, and asterisks denote significant differences (Tukey's *p* < 0.05) between the larval naïve group of a given sex (R) and a larval treatment

The number of circulating hemocytes was altered by larval treatment (Figure 3; treatment *p* < 0.0001). Specifically, female adults that eclosed from larvae that had been infected with *E. coli*, *Enterobacter* sp. or *S. aureus*, or that had been injected with microspheres, had 73%, 63%, 44%, and 48% more circulating hemocytes, respectively, than adults that eclosed from untreated larvae. Likewise, male adults that emerged from those same larval treatment groups had 56%, 58%, 53%, and 35% more circulating hemocytes, respectively, than mosquitoes that arose from naïve larvae. Intriguingly, both female and male mosquitoes that were infected with *E. coli* as larvae but were untreated as adults had 64% and 50% more circulating hemocytes, respectively, than their *E. coli*‐infected adult counterparts that emerged from naïve larvae. Additionally, there was no difference between these individuals (infected as larvae only) and mosquitoes that had been infected as both larvae and adults. Finally, mosquito sex affected the number of circulating hemocytes, with males having fewer hemocytes than females (sex *p* < 0.0001). Thus, these data show that, irrespective of adult treatment, mosquitoes that emerge from immune challenged larvae contain more circulating hemocytes than mosquitoes that emerge from naïve larvae.

### 3.3 Phagocytic activity of individual hemocytes is higher in adults that eclose from infected larvae, especially when adults are re‐infected with the same bacterium

In addition to changes in the abundance of hemocytes, variation in immune proficiency may arise from differences in the frequency or degree to which hemocytes phagocytose invaders (League et al., 2017). Because transstadial transmission of hemocoel infections occurs from larvae to adults (Brown et al., 2018), we first examined untreated adults that eclosed from *E. coli*‐infected larvae. By means of fluorescence microscopy, we could not detect transstadially transmitted *E. coli* within the hemocytes of these mosquitoes; only a dim fluorescent haze without structural definition was observed in fewer than 10% of the hemocytes. This indicates that if these mosquitoes were to be infected as adults, the bacteria observed in the hemocytes would have originated from the inoculum received after eclosion. Having established this, we injected GFP‐expressing *E. coli* into 5‐day‐old adults that had received various treatments as larvae and quantified the phagocytic activity 1 hr later.

**Figure 4:**
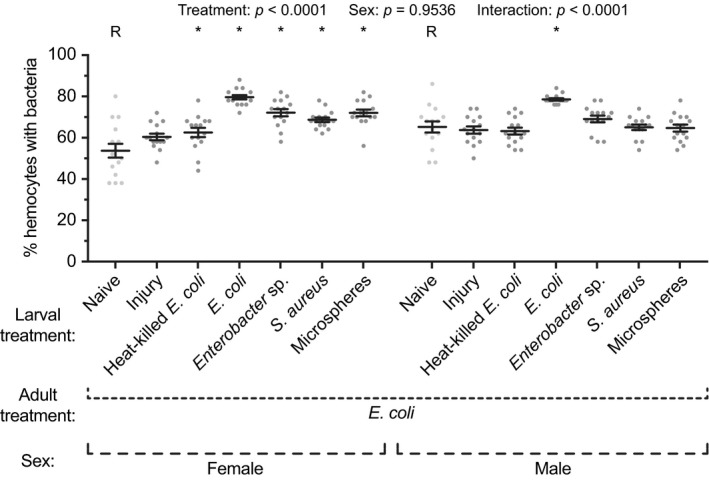
Hemocyte phagocytic index in adult female and male mosquitoes following various larval treatments. Larvae were left unmanipulated (naïve), injured, injected with heat‐killed *E. coli* or microspheres, or infected with *E. coli*, *Enterobacter* sp., or *S. aureus*. Five days after eclosion, the female and male adults were infected with *E. coli*, and the percentage of hemocytes that had phagocytosed *E. coli* was determined 1 hr later. Circles mark the data points, horizontal line marks the mean, whiskers denote the standard error of the mean, and asterisks denote significant differences (Tukey's *p* < 0.05) between the larval naïve group of a given sex (R) and a larval treatment

The phagocytic index—defined as the percentage of hemocytes actively engaged in the phagocytosis of GFP‐*E. coli*—differed significantly between adults from the different larval treatments (Figure 4; treatment *p* < 0.0001). Specifically, the phagocytic index in female adults that emerged from larvae infected with *E. coli*, *Enterobacter* sp., or *S. aureus*, or injected with microspheres, was 48%, 34%, 28%, and 34% higher, respectively, than the phagocytic index in adults that eclosed from untreated larvae. The effect on male adults was milder. Males that emerged from larvae infected with *E. coli* had 18% more hemocytes that had engaged in phagocytosis than mosquitoes that arose from naïve larvae. Differences among the other groups were negligible. Interestingly, although there was no sex‐associated difference in the phagocytic index (sex *p* = 0.9536), there was an interaction between treatment and sex (*p* < 0.0001), which was due to a more pronounced change in the phagocytic index in larval‐infected females relative to their uninfected counterparts.

**Figure 5:**
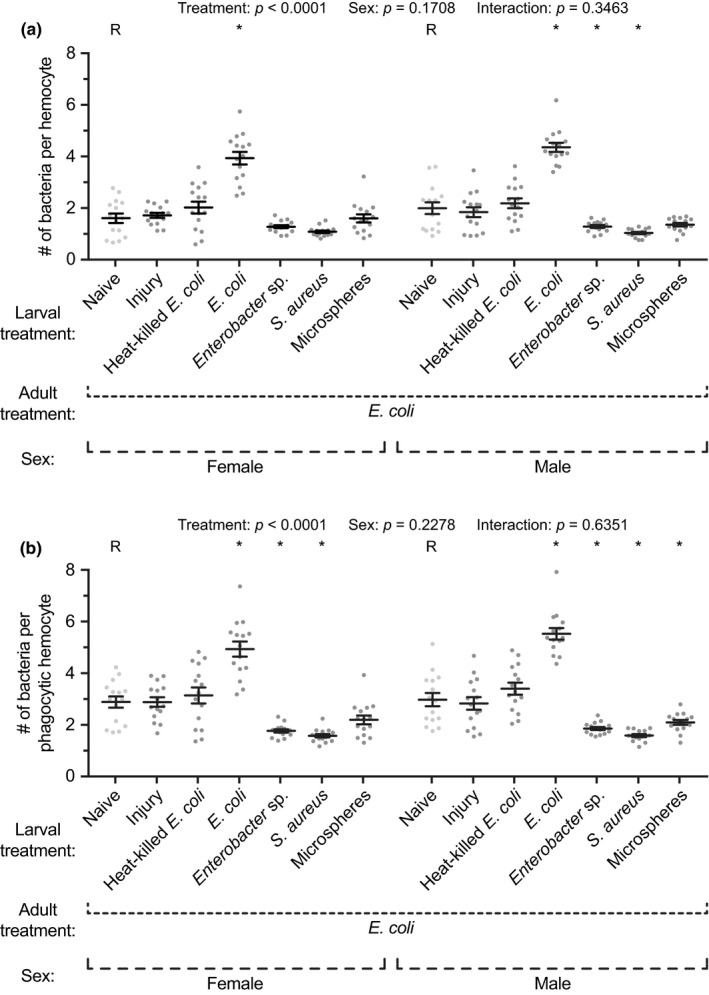
Hemocyte phagocytic capacity in adult female and male mosquitoes following various larval treatments. Larvae were left unmanipulated (naïve), injured, injected with heat‐killed *E. coli* or microspheres, or infected with *E. coli*, *Enterobacter* sp., or *S. aureus*. Five days after eclosion, the female and male adults were infected with *E. coli*, and the number of *E. coli* phagocytosed by each hemocyte (a)—or only the phagocytic hemocytes (b)—was calculated 1 hr later. Circles mark the data points, horizontal line marks the mean, whiskers denote the standard error of the mean, and asterisks denote significant differences (Tukey's *p* < 0.05) between the larval naïve group of a given sex (R) and a larval treatment

The phagocytic capacity—defined as the number of GFP‐*E. coli* phagocytosed by individual hemocytes—also differed significantly between adults that emerged from the different larval groups (Figure 5a; treatment *p* < 0.0001). Specifically, in both female and male adults, the phagocytic response against *E. coli* was significantly stronger when adults emerged from *E. coli*‐infected larvae; these adults had more than twice the phagocytosed bacteria per hemocyte compared to naïve conspecifics, or mosquitoes that emerged from any other treatment group. Interestingly, larval infection with *Enterobacter* sp. or *S. aureus* resulted in a modest reduction in the *E. coli* phagocytic capacity of individual hemocytes. We also calculated the phagocytic capacity for the hemocytes that internalized bacteria (excluding the hemocytes that did not engage in phagocytosis), and the findings were similar to when all hemocytes were considered (Figure 5b). Moreover, the phagocytic capacity of females and males was similar (sex *p* = 0.1708 and *p* = 0.2278 for Figure 5a,b, respectively), and there was no interaction between the effects of treatment and sex (*p* = 0.3463 and *p* = 0.6351 for Figure 5a,b, respectively). Taken together, these data show that a larval infection results in adults that have hemocytes with a higher propensity for killing pathogens via phagocytosis.

### 3.4 Basal expression of immune genes is higher in adults that eclose from infected larvae

Because mosquitoes utilize multiple immune pathways to kill pathogens (Bartholomay & Michel, 2018; Hillyer, 2016), we hypothesized that an infection as a larva leads to increased immune gene expression in adults, relative to adults that emerged from uninfected larvae. To test this, we measured mRNA abundance of several immune genes and a control gene in 5‐day‐old adults that emerged from naïve, injured, or *E. coli*‐infected larvae.

**Figure 6:**
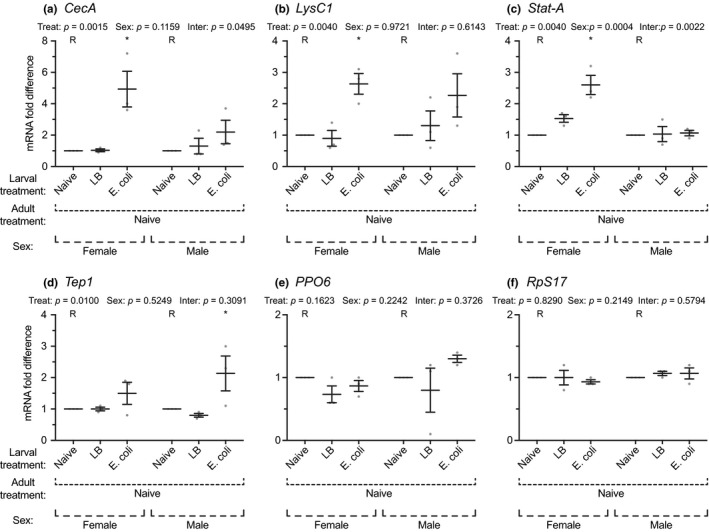
Immune gene expression in adult female and male mosquitoes following various larval treatments. Larvae were left unmanipulated (naïve), injured, or infected with *E. coli*. Five days after eclosion, the expression of *CecA* (a), *LysC1* (b), *Stat‐A* (c), *Tep1* (d), *PPO6* (e), and *RpS17* (f) was measured by qPCR in both female and male adults. *RpS7* was used as the reference gene. Circles mark the data points, horizontal line marks the mean, whiskers denote the standard error of the mean, and asterisks denote significant differences (Tukey's *p* < 0.05) between the larval naïve group of a given sex (R) and a larval treatment

In female mosquitoes, adults that emerged from infected larvae contained 5, 2.6, and 2.6 times more *CecA*, *LysC1,* and *Stat‐A* mRNA than adults that emerged from naïve larvae (Figure 6a–c). Levels of *Tep1*, *PPO6,* and *RpS7* were not significantly affected by a larval infection (Figure 6d–f). In male mosquitoes, the patterns were less pronounced, but adults that emerged from infected larvae contained 2.3 and 2.1 times more *LysC1* and *Tep1* mRNA than adults that emerged from naïve larvae, and a similar but nonstatistically significant trend was observed for *CecA* and *PPO6* (Figure 6a,b,d,e). Levels of *Stat‐A* and *RpS17* were unaffected by a larval infection (Figure 6c,f). In summary, although only a limited number of genes were tested, and there was not perfect congruence between the sexes, adult mosquitoes that emerged from infected larvae had elevated levels of immune gene mRNA, suggesting that these immune effectors are present at higher concentrations at the time an adult receives an infection.

### 3.5 Larval infection does not increase the probability that females survive an infection acquired as an adult

**Figure 7:**
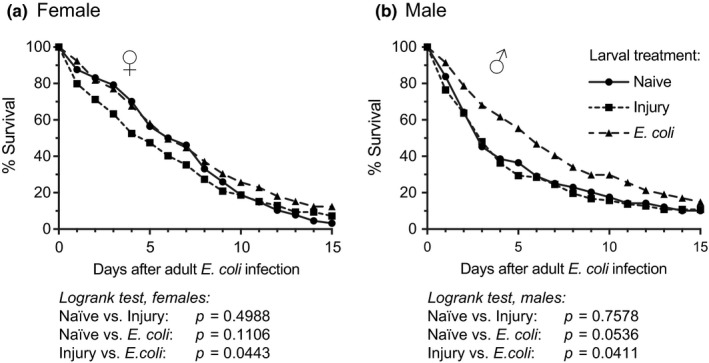
Survival of female and male adult mosquitoes. Larvae were left unmanipulated (naïve), injured, or infected with *E. coli*. Five days after eclosion the female (a) and male (b) adults were infected with *E. coli*, and their survival—reported here as a percentage—was tracked for the next 15 days

Given the infection‐induced passage of bacterial killing proficiency from larvae to adults, we tested whether five‐day‐old adults that emerged from *E. coli‐*infected larvae were better at surviving a bacterial infection than adults that emerged from naïve or injured larvae. Contrary to our expectation, a larval infection did not have a meaningful effect on the ability of females to survive an adult infection (Figure 7a). Although infected female adults that emerged from infected larvae survived marginally better than infected adults that emerged from injured larvae (*p* = 0.0443), their survival was not significantly different from infected adults that emerged from naïve larvae (*p* = 0.1106). For males, the phenotype was stronger (Figure 7b). Infected adults that emerged from infected larvae survived significantly better than those that emerged from injured larvae (*p* = 0.0411). Visual analysis of the data suggested that this was also the case when the comparison was against adults that emerged from naïve larvae. However, because of survival compression toward the end of the experiment (a floor effect), the Logrank *p*‐value did not reach statistical significance (*p* = 0.0536). When the analysis was halted on day 10 to avoid the floor effect, adult males that emerged from infected larvae indeed survived significantly better than those that emerged from naive larvae (*p* = 0.0261). Taken altogether, these data indicate that although a larval infection results in adults that are better able to kill bacteria, this increase in immune proficiency does not necessarily translate into increased long‐term survival when an infection is acquired as an adult.

### 3.6 Larval infection increases the susceptibility of adults to malaria parasites

Having established that a larval infection renders mosquitoes more capable of killing bacteria acquired during adulthood, we set to determine whether this phenotype extended to a malaria infection. To test this, larvae were left unmanipulated (naïve), were injured, or were infected with *E. coli*. After eclosion, adult females were blood fed on mice infected with *Plasmodium yoelii*, and the prevalence and intensity of infection were measured 7–9 days later.

**Figure 8:**
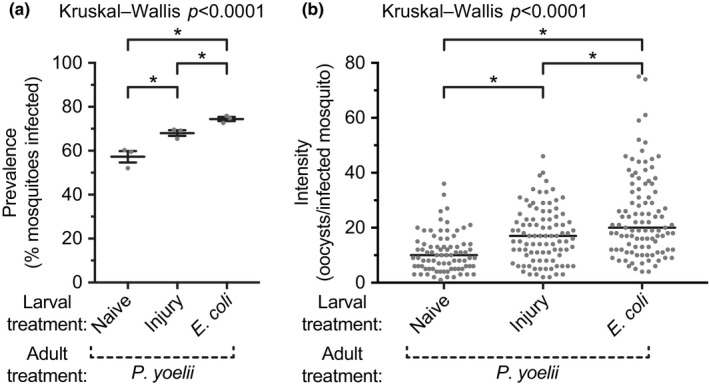
Prevalence and intensity of malaria infection in adult female mosquitoes following various larval treatments. Larvae were left unmanipulated (naïve), injured, or infected with *E. coli*. Five days after eclosion, the adults were blood fed on mice infected with *Plasmodium yoelii*, and the prevalence (a; percent of mosquitoes with oocysts; horizontal line and whiskers denote the mean and the standard error of the mean, respectively) and intensity (b; circles indicate the number of oocysts in infected mosquitoes and the horizontal line marks the median) of infection was measured 7–9 days later. Asterisks denote significant differences (Dunn's *p* < 0.05) between the groups

On average, 75% of mosquitoes that had been infected with *E. coli* as larvae became infected with *P. yoelii* as adults, whereas 68% and 57% of mosquitoes that had been injured or left unmanipulated as larvae, respectively, became infected as adults (Figure 8a; *p* = 0.0036). This indicates that a bacterial infection as larvae renders the eclosed adults more likely to acquire a malaria infection. When parasite intensity in the midgut was measured in mosquitoes that had become infected, the median number of oocysts in adults that eclosed from infected larvae was 140% higher than in adults that eclosed from unmanipulated larvae (Figure 8b; *p < *0.0001). This indicates that even when only the adults that acquired a malaria infection are considered, these infections are much more intense in mosquitoes that eclose from larvae that had been infected with bacteria during their larval stage. The prevalence and infection intensity of mosquitoes that emerged from injured larvae was higher than for mosquitoes that emerged from naïve larvae, but this effect was milder than the effect of a larval infection.

## 4 DISCUSSION

**Figure 9:**
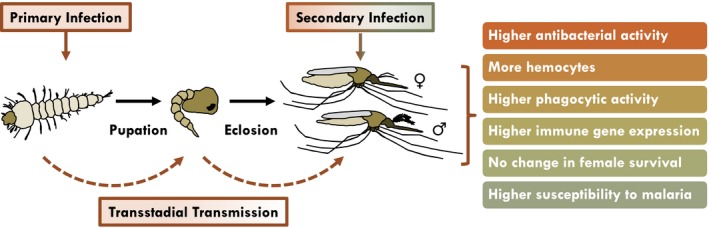
Effect of a larval infection on the immune competency of mosquito adults

Although an infection during the adult stage can activate the immune system such that the mosquito is more responsive to a subsequent infection (Aliota, Chen, Dagoro, Fuchs, & Christensen, 2011; Bargielowski & Koella, 2009; Blanford et al., 2005; Lowenberger et al., 1999; Paul, Nu, Krettli, & Brey, 2002; Rodrigues, Brayner, Alves, Dixit, & Barillas‐Mury, 2010), whether a similar phenomenon occurs across life stages remained largely unexplored. This was a major oversight, as mosquitoes are susceptible to infections during all life stages, and the effects of a larval infection could carry over to impact the probability of pathogen transmission by adult mosquitoes. Here, we show in the African malaria mosquito, *An. gambiae*, that a hemocoelic infection during the larval stage results in transstadial immune activation in a manner that significantly alters the outcome of an infection acquired as an adult, including the outcome of an infection with malaria parasites (Figure 9).

Our initial experiments showed that adult mosquitoes that emerge from immune challenged larvae are more resistant to a bacterial infection than adults that emerge from naïve or injured larvae. This heightened immune proficiency in adults occurs regardless of whether the bacterial infection is the same or different from the infection acquired as larvae. These data support our initial hypothesis that a larval hemocoelic infection enhances the immune response of the resulting adult. Additionally, these findings are consistent with studies that focused on a single anopheline life stage, whereby a previous infection acquired as an adult can render those individuals better equipped to fight a later infection (Lowenberger et al., 1999; Rodrigues et al., 2010). Finally, these data support findings that environmental exposure of mosquito larvae—mainly culicine larvae—to different microorganisms can have a negative effect on adult‐acquired pathogens (Dickson et al., 2017; Kala & Gunasekaran, 1999; Mahapatra, Hazra, Rup, Acharya, & Dash, 1999; Moreno‐Garcia et al., 2015; Paily, Geetha, Kumar, & Balaraman, 2012; Ramirez et al., 2014). Taken together, larval exposure to certain microorganisms, be it in the gut or hemocoel, can boost the immune system of an adult mosquito. The underlying drivers behind this enhanced immunity remain largely unknown, but a larval hemocoelic infection can be transstadially passaged to adults, which may increase immune alertness due to a persistent infection (Brown et al., 2018). This idea is further supported by our finding that a larval challenge with an undegradable immune elicitor (microspheres) increases bacterial clearance in adults, whereas larval challenge with a degradable immune elicitor (heat‐killed *E. coli*) does not.

Because hemocytes are the primary responders to an infection in the hemocoel (Hillyer & Strand, 2014), we hypothesized that a larval infection increases the number of hemocytes in adult mosquitoes. This hypothesis was based on two observations. First, naïve larvae have more hemocytes than naïve adults (League et al., 2017). Second, the number of hemocytes in adult mosquitoes declines with age, a phenomenon that contributes to immune senescence (Castillo, Robertson, & Strand, 2006; Hillyer et al., 2005; King & Hillyer, 2013; League et al., 2017; Pigeault, Nicot, Gandon, & Rivero, 2015). However, an adult infection results in an increase in the number of hemocytes in some mosquito species but not others (Coggins et al., 2012; Hillyer et al., 2005; King & Hillyer, 2013; League et al., 2017). When infection—or blood feeding—induces an increase, this is because of mitosis by circulating hemocytes, although sessile hemocytes may also release and enter circulation (Bryant & Michel, 2014; Castillo, Brown, & Strand, 2011; King & Hillyer, 2013; Sigle & Hillyer, 2016, 2018). In the present study, we found that mosquitoes that receive both a larval infection and an infection as an adult have more hemocytes than those that were not infected as larvae. Additionally, the number of hemocytes in mosquitoes that received an infection at both the larval and adult stages is no different from the number in mosquitoes that only received a larval infection. This indicates that the increase in hemocytes is primarily due to hemocyte replication following the larval infection instead of rapid hemocyte replication following an adult infection. An alternative explanation is that the carryover of a larval infection into adulthood induces a persistent and elevated rate of hemocyte mitosis.

Hemocytes kill bacteria via several mechanisms, one of which is phagocytosis (Hillyer et al., 2005; Hillyer & Strand, 2014; League et al., 2017). Phagocytosis has been implicated in immune priming of several insect species (Pham, Dionne, Shirasu‐Hiza, & Schneider, 2007; Roth & Kurtz, 2009; Wu, Li, Liu, Ding, & Yi, 2015). Thus, we hypothesized that a larval infection activates the immune system such that when adults receive a later infection the phagocytic proficiency of circulating hemocytes is elevated. We found that a larval infection leads to a modest increase in the percentage of adult hemocytes that engage in phagocytosis, and that when the larval and adult infections are the same, the number of *E. coli* phagocytosed by each hemocyte is also increased. However, a larval infection with *S. aureus* or *Enterobacter* sp. modestly reduces the number of *E. coli* that are phagocytosed by the hemocytes of adults. Although some of these data may seem counterintuitive, a larval infection with any bacteria results in an elevated number of hemocytes. Thus, with more hemocytes circulating in the hemolymph—and presumably more sessile hemocytes as well—each hemocyte may need to do less to control the infection. The present findings agree with results from a comparative analysis of immune responses in larval and adult anopheline mosquitoes (League et al., 2017). Larvae are immunologically stronger than adults, yet the phagocytic index and phagocytic capacity are lower in larvae than in adults. Furthermore, hemocytes produce numerous humoral factors with antibacterial activity (Bartholomay et al., 2004; Baton, Robertson, Warr, Strand, & Dimopoulos, 2009; De Das et al., 2018; Pinto et al., 2009; Severo et al., 2018; Smith et al., 2016), and hence, components of the humoral response may reduce the burden on cellular immunity. We found that this indeed is the case. Female adults that emerged from infected larvae had elevated levels of *CecA*, *LysC1,* and *Stat‐A* mRNA, and comparable trends—albeit not perfectly congruent—were found in males. These data suggest that increased levels of immune effectors are present in the hemocoel of adults that emerge from infected larvae, such that these individuals can respond more swiftly to infection.

Because of the enhanced antibacterial response observed in adults from immune challenged larvae, we predicted that a larval infection results in adults that are better able to survive a systemic bacterial infection. We found that although infected adults that eclosed from infected larvae tended to survive better than infected adults that eclosed from injured larvae, larval infection improved the ability of males—but not females—to survive a bacterial infection acquired as an adult. The reason behind the lack of an obvious survival benefit for females is not clear, but it is possible that the effect of the larval infection is masking the protection conferred to adults. We recently reported that a larval infection negatively impacts the survival of untreated adults (Brown et al., 2018). Thus, the baseline survival is lower in adult mosquitoes that encountered an infection as larvae, and hence, a larval infection could be providing a modicum of protection against an adult infection but in this experiment this effect is undetectable. That said, it is also possible that different outcomes would have been observed following infection with different pathogens or different doses, as has been observed within the mosquito adult life stage and between life stages (Hillyer et al., 2005; League et al., 2017; Rhodes, Thomas, & Michel, 2018). Together, these findings suggest that there are trade‐offs between heightened immune activity and other physiological processes.

We then examined whether a larval infection impacts the ability of mosquitoes to harbor malaria parasites, and contrary to our expectations, adult mosquitoes that imbibe a blood meal containing *P. yoelii* are more likely to become infected if they emerged from larvae that had received a hemocoelic bacterial infection. A larval infection also increases the severity of an adult‐acquired malaria infection. These findings were particularly surprising for two reasons. First, hemocytes are positive modulators of the anti‐*Plasmodium* immune response in the midgut (Kwon & Smith, 2018; Lombardo & Christophides, 2016; Pinto et al., 2009; Smith et al., 2016). Second, many antibacterial effectors that are produced by hemocytes also have anti‐*Plasmodium* activity, including two of the genes that showed larval infection‐induced regulation as adults: *CecA* and *Tep1* (Blandin et al., 2004; Dong et al., 2011; Kim et al., 2004). But this is not universal; *Stat‐A* has different effects on early and late *Plasmodium* development, and *LysC1*, although having antibacterial activity, is a positive regulator of malaria parasite infection (Gupta et al., 2009; Kajla, Andreeva, Gilbreath, & Paskewitz, 2010; Kajla et al., 2011). However, in this study the compartments infected by malaria parasites and *E. coli* were different—*Plasmodium* infection was measured in the midgut, whereas bacterial infection was measured in the hemocoel—and perhaps the necessity of simultaneously having to fight a persistent infection in the hemocoel and a midgut infection is too much for a mosquito to overcome. Alternately, components in the vertebrate blood that modulate a myriad of physiological processes—including the mosquito immune system (Pakpour, Riehle, & Luckhart, 2014)—may significantly affect the outcome of co‐infection.

Sex‐specific differences in immunity have been reported in other insects (Duneau et al., 2017; Jacobs et al., 2016; Mitaka, Kobayashi, & Matsuura, 2017). However, the present study is unique in that it examined immune parameters in both female and male mosquitoes, which differs from what has historically been an almost exclusive focus on the immune system of females. Although conducting comparisons between the sexes was not a major driver of the design of this study, sex‐specific differences were detected in most experiments. Mainly, immune proficiency is stronger in females in that they exhibit increased bacterial killing capacity, have twice the number of hemocytes, and express higher levels of immune effector genes. However, the increased immune proficiency in female mosquitoes does not translate into a markedly increased ability to survive an infection.

Finally, the phenotype observed here is often considered to be a consequence of immune priming, and in this specific case, transstadial immune priming. Although transstadial immune priming may be occurring, we know that a hemocoelic bacterial infection is transstadially transmitted to the adults (Brown et al., 2018). Thus, we elected to instead refer to the phenotype observed here as transstadial immune activation because immune priming, in its strictest sense, involves the challenge of a quiescent immune system that has been primed in the past (Melillo et al., 2018).

## 5 CONCLUSIONS

In summary, this study assessed the effect of a larval infection on the ability of female and male mosquitoes to fight a subsequent infection as adults. We found evidence for transstadial immune activation in that a larval infection results in adults with more hemocytes, elevated immune gene expression, and a greater ability to kill bacteria in their hemocoel (Figure 9). However, protection is limited; a larval infection does not meaningfully affect the probability that adult females survive a bacterial infection and results in increased susceptibility to a malaria parasite infection.

## CONFLICT OF INTEREST

The authors declare that they have no competing interests.

## AUTHOR'S CONTRIBUTIONS

LDB and JFH conceptualized the study. LDB, LLMS, and JFH designed the experiments. LDB, LLMS, GAT, and TYE‐L conducted the experiments. LDB, LLMS, GAT, TYE‐L, and JFH analyzed the data. LDB and JFH wrote the manuscript. All authors read and approved the manuscript.

## DATA AVAILABILITY STATEMENT

All relevant data are presented in the paper.
